# A Randomized, Controlled Trial of Treat-and-Extend vs. Pro Re Nata Regimen for Neovascular Age-Related Macular Degeneration

**DOI:** 10.3389/fmed.2022.852519

**Published:** 2022-06-20

**Authors:** Huixun Jia, Bing Lu, Yuanzhi Yuan, Fei Yuan, Lei Li, Yanping Song, Ao Rong, Minwen Zhou, Fenghua Wang, Xiaodong Sun

**Affiliations:** ^1^National Clinical Research Center for Ophthalmic Diseases, Shanghai, China; ^2^Department of Ophthalmology, School of Medicine, Shanghai General Hospital (Shanghai First People’s Hospital), Shanghai Jiao Tong University, Shanghai, China; ^3^Shanghai Key Laboratory of Fundus Diseases, Shanghai, China; ^4^Shanghai Engineering Center for Visual Science and Photomedicine, Shanghai, China; ^5^Department of Ophthalmology, Zhongshan Hospital, Fudan University, Shanghai, China; ^6^Department of Ophthalmology, Fudan University Eye and ENT Hospital, Fudan University, Shanghai, China; ^7^Department of Ophthalmology, Central Theater Command General Hospital, Wuhan, China; ^8^Department of Ophthalmology, Tongji Hospital, Tongji University School of Medicine, Shanghai, China

**Keywords:** anti-VEGF, treat-and-extend, pro re nata, neovascular age-related macular degeneration, non-inferiority study

## Abstract

**Purpose:**

To compare the efficacy and safety of conbercept using a treat-and-extend (T&E) regimen vs. a pro re nata (PRN) regimen in Chinese patients with neovascular age-related macular degeneration (nAMD).

**Methods:**

This was a randomized, multicenter, non-inferiority study. After an initial loading phase of three consecutive monthly intravitreal injections of 0.5 mg Conbercept, the patients were treated to PRN or T&E regimen. The prespecified retreatment criteria was defined as a more than 5-letter decrease in BCVA from the previous visit or any evidence of new retinal hemorrhages, or the presence of any IRF and any SRF of more than 200 μm in height at the sub-foveal center. The primary outcome was the mean change in best-corrected visual acuity (BCVA) from baseline to 24 months, with a prespecified non-inferiority limit of −5 letters.

**Results:**

From July 2016 through August 2018, 141 participants were allocated and treated (T&E, *n* = 69; PRN, *n* = 72). About one fifth of the overall participants were dropped out during the 12-month follow-up (28/141, 19.9%), and about one thirds of the overall participants were lost during the 24-month follow-up (51/141, 36%). At 2 years, mean BCVA letter improvement was + 4.0 in the T&E group vs. + 5.1 in the PRN group, and T&E regimen was not non-inferior to PRN regimen [difference, −1.169 letters; 95% confidence interval (CI): −6.864 ∼ 4.526]. Subgroup analyses also demonstrate the similar results in PCV patients, naive patients and no-naive patients. The mean decrease in central subfield thickness were 180 ± 165 μ*m* in the T&E group and 247 ± 230 μ*m* in the PRN group, respectively. The patients in the PRN group had required significantly fewer injections than those in the T&E group (12.4 vs. 14.6 injections, *P* = 0.041). The types and rates of adverse events were comparable in the two treatment groups.

**Conclusion:**

These findings suggest that the T&E regimen was not non-inferior to the PRN regimen in patients with nAMD in terms of BCVA outcomes through 24 months.

**Clinical Trial Registration:**

ClinicalTrials.gov, identifier NCT02802657.

## Introduction

Neovascular age-related macular degeneration (nAMD) is a chronic, progressive disease and a leading cause of irreversible vision loss (1). The introduction of anti-vascular endothelial growth factor (anti-VEGF) agents in ophthalmic clinical practice has revolutionized the treatment of nAMD (2). Timely treatment with anti-VEGF agents facilitates improving or maintaining visual acuity (VA) over a certain period. In the MARINA and ANCHOR trials, intravitreal ranibizumab injected monthly was reported to significantly improve mean visual acuity compared with sham treatment and photodynamic therapy (PDT) (3, 4).

Despite substantial progress, the need for monthly intravitreal injections imposes a burden on patients due to the cost and inconvenience. To reduce this burden, a pro re nata (PRN) regimen with treatment-as-needed and monthly visit was introduced. Two such studies, IVAN and CATT, evaluated improvements in best-corrected visual acuity (BCVA) in PRN schedules vs. monthly dosing schedules, with monthly follow-ups of patients and found the PRN schedule was non-inferior to monthly dosing schedule (5, 6). Although the PRN regimen can reduce the injection frequency and economic burden for treating nAMD, the cost and inconvenience associated with frequent visits persist. Of note, the HORIZON trial showed an incremental decline of the visual acuity gains among patients who were not strict to monthly visit (7).

To address this issue, a treat-and-extend (T&E) regimen was proposed, with the aim of extending visits and treatment intervals once nAMD was stabilized via monthly injections. This regimen is now commonly used in ophthalmology practice (8). To demonstrate the true value of this T&E regimen in nAMD populations, vision outcomes achieved using this regimen must be head to head compared with those obtained using the PRN regimen.

Conbercept (Lumitin; Chengdu Kanghong Biotech Co., Ltd., Chengdu, China), a recombinant fusion protein, contains the second Ig-like domain of VEGFR-1 and the third and fourth Ig-like domains of VEGFR-2, was fused to the Fc portion of human IgG1. It has high binding affinity to all isoforms of VEGF and placental growth factors (PlGF), and a long half-life in vitreous. Previous studies demonstrated that conbercept can successfully improved vision outcomes in nAMD patients (9). The purpose of this study was to evaluate the efficacy and safety of the T&E and PRN regimen of conbercept for nAMD patients in Chinese populations, specifically to demonstrate the non-inferiority of the T&E regimen as compared with the PRN regimen (clinicaltrials.gov; identifier NCT02802657).

## Materials and Methods

### Patients

From July 2016 through August 2018, we enrolled 141 patients with a diagnosis of nAMD at five teaching and general hospitals in China. The study protocol was reviewed and approved by an independent ethics committee at each center. All the patients provided written informed consent. All the patients underwent baseline ophthalmic examinations, including a slit-lamp examination, tonometry, fundus examination, BCVA (ETDRS letter score), spectral-domain optical coherence tomography (SD-OCT), fluorescein angiography (FA), and indocyanine green angiography (ICGA).

### Eligibility Criteria

The eligibility criteria included aged ≥ 50 years; active choroidal neovascularization lesions, including classic and occult, comprising > 50% of the total lesion area, as assessed by FA; intraretinal fluid (IRF) and/or subretinal fluid (SRF) affecting the central subfield, as assessed on SD-OCT; BCVA between 20/30 and 20/320; and no fibrosis or geographic atrophy affecting the central subfield. Final anatomic eligibility determination was made by the Shanghai Reading Center.

### Treatments

**FIGURE 1:**
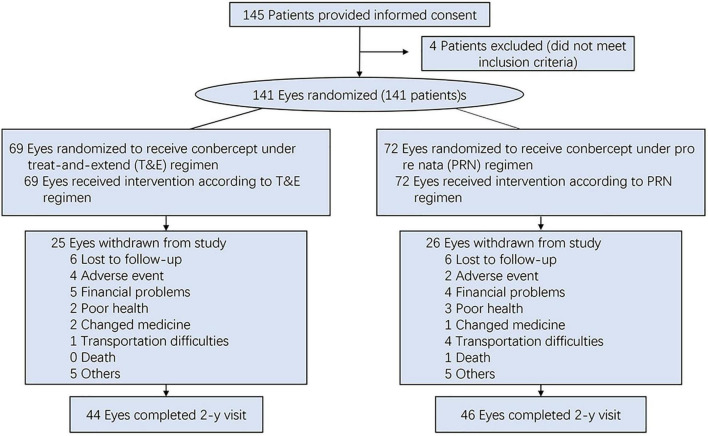
The flowchart of the study.

The patients were randomized to one of two treatment arms: a PRN regimen (*n* = 72) or a T&E (*n* = 69). After an initial loading phase of three consecutive monthly intravitreal injections of 0.5 mg Conbercept, the patients were treated according to the randomization treatment arm (Figure 1). Patients receiving PRN treatment, with monthly visit and treatment-as-needed, were re-treated if the study eye met the prespecified disease activity criteria. In the T&E arm, after the 3 monthly loading phase, if disease stability was achieved, the interval between injections was extended by a further 2 weeks, to a maximum of 12 weeks. In cases of evidence of disease activity, the treatment interval was shortened by 2 weeks to a minimum of 4 weeks. Disease activity criteria defined as a more than 5-letter decrease in BCVA from the previous visit or any evidence of new retinal hemorrhages, or the presence of any IRF and any SRF of more than 200 μm in height at the sub-foveal center (10).

### Endpoints

The prespecified primary endpoint was the mean change in BCVA between 2 years and baseline. The secondary outcomes included changes in central subfield thickness (CST) on OCT, the number of injections, and the incidence of treatment-related adverse events. The BCVA assessors and the imaging technicians were masked to regimens allocation.

### Statistical Analysis

Sample size calculations were based on the change in BCVA from baseline to 24 months, non-inferiority margin of −5 letters, assuming a true difference of −2.5 letters between the two groups, and a standard deviation (SD) for changes in VA of 5 letters. We determined that a sample of 140 patients would provide a power of 85%, at a one-sided significance level of 0.025. Primary analyses were performed on the basis of both intention-to-treat (ITT) principle and on-treatment-analyses. Results are presented as means and standard deviations for continuous variables and counts and percentages for categorical variables. Comparisons between two groups were performed using Pearson’s chi-square test (discontinuous variables) or the Student’s *t*-test (continuous variables). Non-inferiority was accepted as the lower boundary of the confidence interval (CI) for an estimated (mean) treatment difference of above −5 letters. Statistical analyses were performed using SPSS software version 19.0 (SPSS, Inc., Chicago, IL).

## Results

As of August 2020, there were 141 enrolled patients with a mean age of 72.3 (SD, 7.5) years enrolled in the study (Table 1). Of these, there were 51 (36%) females, and 46 (33%) of the patients were polypoidal choroidal vasculopathy (PCV) (Table 1). Of the 141 patients, 113 (80.1%) completed the 12-month follow-up, and 90 (64%) completed the 24-month follow-up. According to the efficacy analysis of the ITT, the primary endpoint was not met, with non-inferiority was not demonstrated for the T&E regimen (4.0 ± 17.8 letters) compared to the PRN regimen (5.1 ± 16.3 letters). The difference was −1.169 (95%CI: −6.864 ∼ 4.526) (Table 2). In both treatment groups, the mean BCVA increased from baseline during the first 3 months of treatment by 5 letters, with a subsequent steady increase of 1∼3 letters over the following 20 months. At 24 months, BCVA improved in both treatment groups (Supplementary Figure 1). A gain of 15 or more letters was achieved by 20% of those in the T&E group and 28% of those in the PRN group (Supplementary Table 1). In the subgroup analyses, T&E regimen was also not non-inferior to PRN regimen in the PCV, SRF permissive, treatment-naive or treatment-experienced subgroups (Supplementary Table 2 and Supplementary Figure 2).

**TABLE 1:** Baseline characteristics between T&E and PRN group.

	T&E (*n* = 69)	PRN (*n* = 72)	*P*-value
Age (years) ± SD	73.0 ± 7.1	71.9 ± 7.9	0.305
**Sex, No. (%)**			
Male	41 (59.4)	49 (68.1)	
Female	28 (40.6)	23 (31.9)	
BCVA (ETDRS letters) ± SD	53.0 ± 14.0	51.9 ± 13.7	0.649
Central subfield thickness (μm) ± SD	543.1 ± 168.2	581.1 ± 244.7	0.328
Type of CNV			0.303
Type 1 NV	19	18	
Type 2 NV	17	17	
Mixed type NV	15	9	
PCV	18	28	
History of anti-VEGF therapy			0.456
Yes	19	16	
No	50	56	
History of diabetes mellitus			0.883
Yes	9	10	
No	60	62	
History of hypertension			0.696
Yes	38	42	
No	31	30	

*ETDRS, Early Treatment of Diabetic Retinopathy Study; BCVA, Best-Corrected Visual Acuity; T&E, treat-and-extend; PRN, pro re nata.*

**TABLE 2:** The 12 and 24 months BCVA change from baseline in ITT analyses.

BCVA mean change	T&E (*n* = 69)	PRN (*n* = 72)	Difference (95% CI)
By the end of 24 months	4.0 ± 17.8	5.1 ± 16.3	−1.169 (−6.864∼ 4.526)
By the end of 12 months*	6.1 ± 17.8	10.4 ± 12.4	−4.343 (−9.455∼0.769)

*BCVA, Best-Corrected Visual Acuity; T&E, treat-and-extend; PRN, pro re nata.*

*Missing data were imputed using the last observation carried forward method.

The mean CST was significantly reduced in both the treatment regimen groups. According to the ITT analysis, there was no significant between-group difference in the CST at 24 months, with a mean decrease in the CST of 180 ± 165 μ*m* in the T&E group and 247 ± 230 μ*m* in the PRN group (*P* = 0.135) (Supplementary Table 4).

The mean (SD) number of anti-VEGF injections between randomization and 24 months (i.e., the last injection prior to measurement of the primary outcome) was 14.6 (4.1) in the T&E group and 12.4 (6.1) in the PRN group (*P* = 0.041). In the T&E arm, the treatment interval was able to be extended to 8 or more weeks for 46 (66.7%) of the participants, and 25 (36.2%) participants reached the maximum 12-week extension interval. At 24 months, the number of visits in the T&E group (*n* = 15.4) was lower than that in the PRN group (*n* = 23.1) (Table 3). There were 90 participants reached the visit at 2 years, 46 participants in PRN group and 44 participants in T&E group were included in the on-treatment-analysis. For 24-month BCVA, T&E was neither non-inferior nor inferior to PRN, which were similar to the ITT analyses (Supplementary Table 3 and Supplementary Figure 3).

**TABLE 3:** The number of injections and visits at 24 months.

Factors	T&E (*n* = 44)	PRN (*n* = 46)	*P*-value
Total number of injections at 24 months, mean	14.6 ± 4.1	12.4 ± 6.1	0.041
Total number of visits at 24 months, mean	15.4 ± 3.8	23.1 ± 3.5	<0.001

*T&E, treat-and-extend; PRN, pro re nata. The numbers of injections and visits were calculated using the cases who completed the 24-months follow-up.*

Overall, 105 patients (74.5%) experienced at least 1 adverse event (AE) during the study [55 patients (79.7%) in the T&E group and 50 patients (69.4%) in the PRN group]. At least 1 non-ocular serious adverse event (SAE) during the study was reported by 4 patients (6%) in the T&E group and 3 patients (4%) in the PRN group. One was death in a car accident in PRN group (Table 4). None was suspected by the investigator to be related to treatment.

**TABLE 4:** Systemic and ocular adverse events of interest through 2 years of follow-up.

Events	T&E	PRN
**Systemic adverse events, No. (%)**	**4**	**3**
Cerebral infarction	0	1
Tumor operations	1	1
Infectious diseases	2	0
Other surgeries	1	0
Death from any cause	0	1
**Ocular adverse events, No. (%)**	**3**	**1**
Vitreous hemorrhage	2	1
Macular hole	1	0

*T&E, treat-and-extend; PRN, pro re nata.*

## Discussion

In this multicenter, randomized, controlled trial (RCT), the T&E regimen with Conbercept did not meet the primary objectives to demonstrate non-inferiority (margin: 5 letters) to PRN in terms of changes in BCVA from baseline to 24 months. Although T&E and PRN with VEGF inhibitors are commonly used regimens for treating nAMD in clinical settings (11). To our knowledge, only one head-to-head RCT (In-eye trial) evaluated long-term BCVA outcomes in naive nAMD patients treated with T&E ranibizumab or PRN ranibizumab (12). The authors of the In-eye trial concluded that T&E was non-inferior to PRN at a 12-month follow-up. However, based on data presented in their study, the mean difference in BCVA between the T&E and PRN arms was −3.34 letters (95%CI: −7.83 to 1.15). The lower limit in their study exceeded the prespecified non-inferiority margin of 5 letters. In fact, the In-eye trial did not demonstrate non-inferiority of the T&E regimen, which was consistent with the results of our study.

One should notice that up to 33% of the study population were PCV in our study due to the prevalence in Asian patients varies markedly with Caucasian patients (13). Although, recent studies have shown satisfactory effects of ranibizumab and aflibercept in patients with PCV (14–18), whether T&E regimen is suitable for PCV is controversial. Only a retrospective study reported an effectively BCVA improvement in patients with PCV treated by the T&E regimen using ranibizumab (19). However, this finding must be interpreted with caution, as 23.5% of the PCV patients were non-responders to ranibizumab and were removed from the statistical analysis. Our PCV subgroup analysis result showed that the T&E treatment regimen was also not meet non-inferiority in comparison to the PRN regimen with conbercept injection. PCV is a high heterogeneity subtype of nAMD, and its polypoidal lesions and associated branching vascular network complex often persist after anti-VEGF treatment, resulting in the risk of recurrent bleeding and poor outcome. The EVERST-II study also confirmed that combination therapy with PDT was superior to anti-VEGF monotherapy in terms of the gain in BCVA and polyp closure for treatment PCV (20). Hence, based on the literature and the findings of the present study, we believed that T&E agent was not advisable for the PCV patients. PCV may require more aggressive treatment regimen, and those non-responders should be treated by additional means such as PDT or other anti-VEGF agents.

Another concern is that a high tolerance of SRF (SRF > 200 μm) is considered as the retreatment criteria in our study. The rational we consider for this criterion were: (1) FLUID study showed patients treated with T&E protocol who tolerated some SRF (SRF < 200 μm) achieved VA that is comparable, with fewer injections, with that achieved when treatment aimed to resolve all SRF completely (10); (2) HARBOR Study found that residual SRF would be beneficial by reducing rates of atrophy in nAMD (21). According to our findings, the mean change in BCVA and the mean frequency of anti-VEGF injections was lower compared to previous study without permissive SRF (15). And the subgroup analyses of SRF permissive and PCV in our study showed T&E regimen were also non-inferior to PRN regimen at the 24th month, which is consistent with our main conclusion. However, the improved BCVA, due to the treatment in the first year, could not to be maintained at the end of the second year in SRF permissive and PCV patients with either T&E or PRN regimens. Possible reasons for this phenomenon were that patients with more tolerated SRF were accepted and up to 30% PCV patients were included in our study. Hence, more tolerated SRF should be carefully considered before taking it as a re-treated standard, especially for PCV patients.

Over the course of our study, the incidence of ocular SAEs and non-ocular SAEs were similar. Overall, the safety findings were consistent with the previous safety profiles of aflibercept and ranibizumab (22). It is worth noting that in the T&E group, vitreous hemorrhages occurred in two patients with PCV when the treatment interval was extended to 6 and 8 weeks, respectively. Previous research suggested that pulsatile polyps tended to rupture and cause severe hemorrhages (23). A sudden hemorrhage may occur in patients with residual polyps after discontinuation of anti-VEGF therapy, with several studies reporting recurrence rates as high as 40–78.6% after 3 years (24, 25). These results further indicate that shorter follow-up intervals are necessary in patients with PCV and that the T&E regimen might not be suitable for patients with PCV.

Limitations of our study warrant mention. In our study, rescue therapy, such as PDT, was not included in the study protocol, which might have led to worse visual outcomes in the patients with PCV who did not respond to conbercept. In addition, our study had a relatively high dropout rate in the second year due to the outbreak of COVID-19. Thus, these missing data were calculated using the last observation carried forward method (26). Lastly, when the generalization of findings from the current study to other forms of anti-VEGF agents, careful considerations should be taken, due to the fact that there is a lack of external validity.

## Conclusion

In conclusion, among eyes with nAMD, treatment with conbercept T&E regimen resulted in the improvements of BCVA that was not non-inferior to PRN regimen at 2 years. Although large-scale studies with longer term follow-up are needed, T&E may be not an advisable treatment regimen for the Chinese nAMD patients, especially for PCV patients.

## Data Availability Statement

The data that underlie the results reported in this article will be made available to individual researchers on reasonable request by contacting the corresponding author.

## Ethics Statement

The studies involving human participants were reviewed and approved by the ethics committee at each center. The patients/participants provided their written informed consent to participate in this study. Written informed consent was obtained from the individual(s) for the publication of any potentially identifiable images or data included in this article.

## Author Contributions

XS: full access to all the data in study and takes responsibility for the integrity of the data and the accuracy of data analysis. HJ: statistical analysis. BL, HJ, and XS: administrative, technical, or material support. FW and XS: supervision. All authors: concept and design, acquisition, analysis, interpretation of data, drafting of the manuscript, and critical revision of the manuscript for important intellectual content.

## Conflict of Interest

The authors declare that the research was conducted in the absence of any commercial or financial relationships that could be construed as a potential conflict of interest.

## Publisher’s Note

All claims expressed in this article are solely those of the authors and do not necessarily represent those of their affiliated organizations, or those of the publisher, the editors and the reviewers. Any product that may be evaluated in this article, or claim that may be made by its manufacturer, is not guaranteed or endorsed by the publisher.

## Supplementary Material

Supplementary Figure 1 The mean BCVA change from baseline to 24 months in both treatment groups.

Supplementary Figure 2 The BCVA examined at baseline, 12 and 24 months using T&E and PRN regimen for SRF permissive patients **(left)** and PCV patients **(right)**.

Supplementary Figure 3 Comparisons of BCVA observed from patients treated with T&E and PRN regimen (only patients who completed the 24-months follow-up were included, *n* = 90).
