# Supplementary figures and images for: A Randomized, Controlled Trial of Treat-and-Extend vs. Pro Re Nata Regimen for Neovascular Age-Related Macular Degeneration
Source: Front Med (Lausanne). 2022 Jun 20;9:852519. doi: 10.3389/fmed.2022.852519 (PMC9251380; doi:10.3389/fmed.2022.852519)

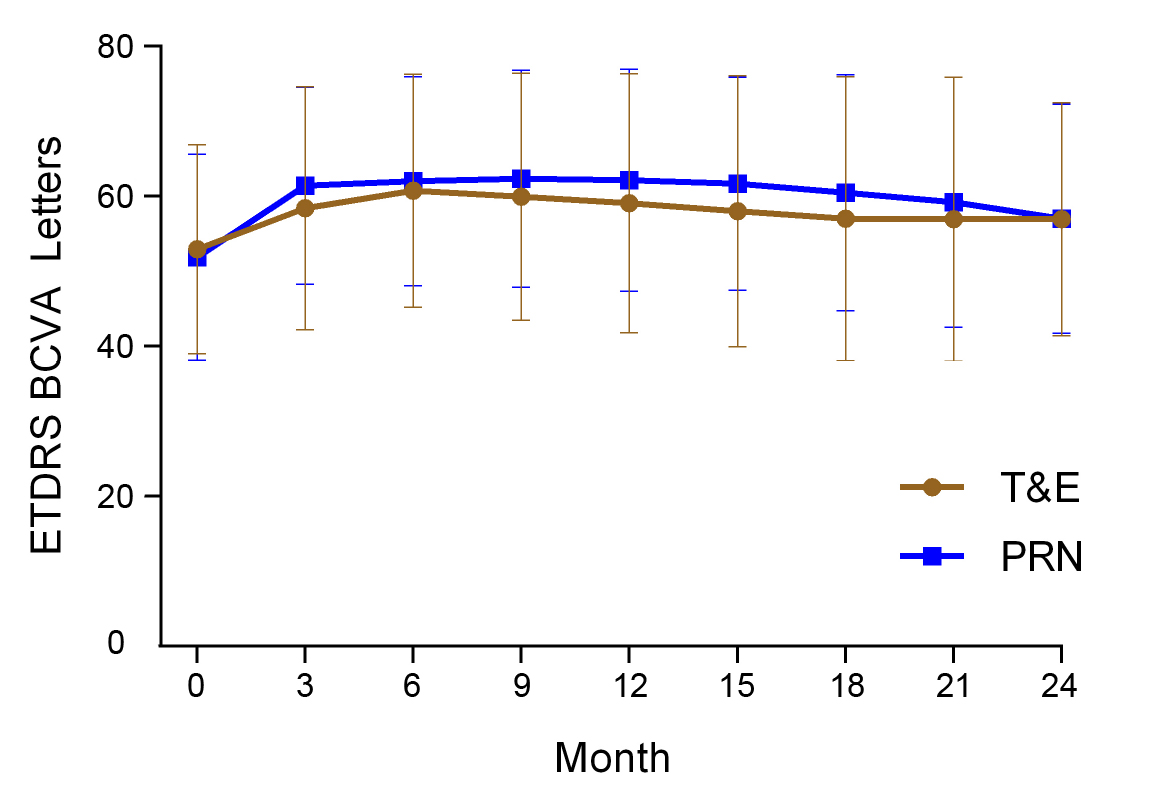

Supplement: Supplementary Figure 1 — The mean BCVA change from baseline to 24 months in both treatment groups. [file Figure_1.tif]

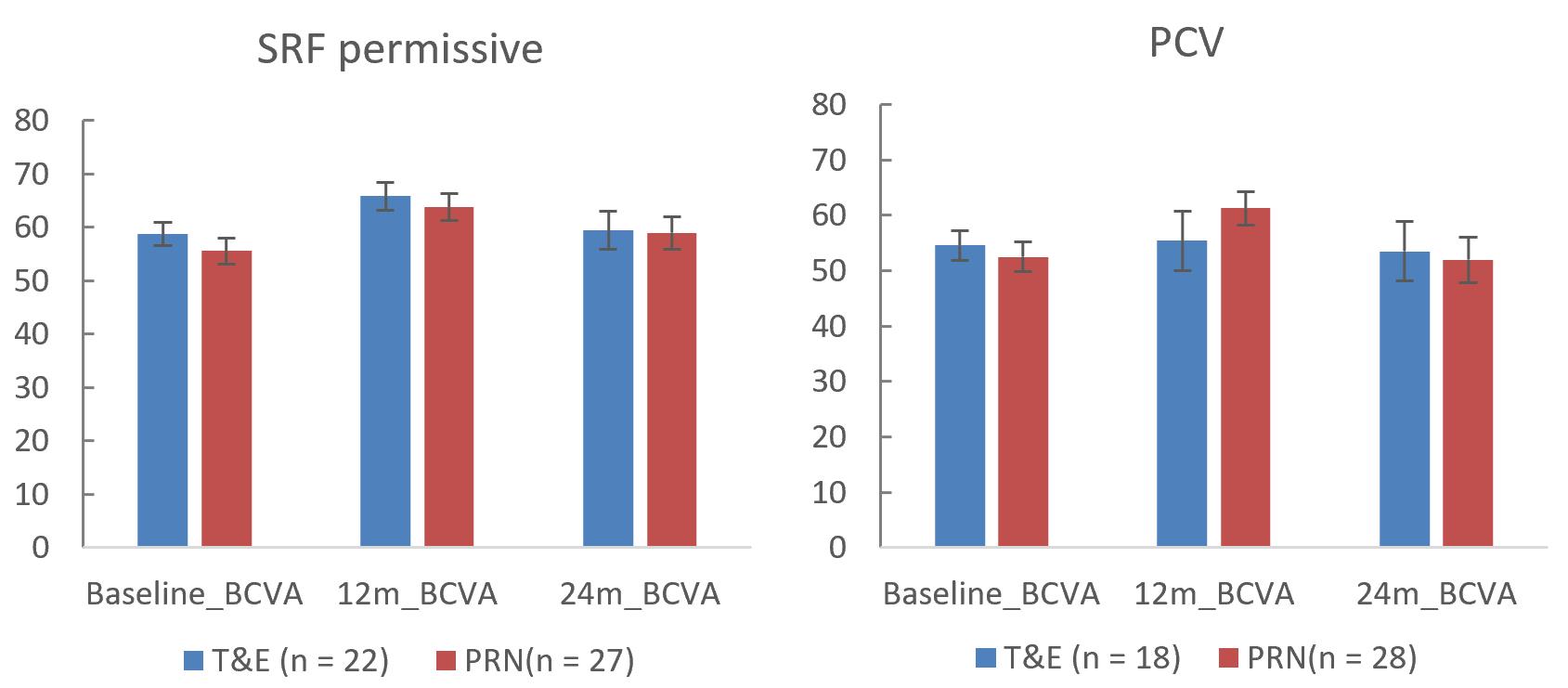

Supplement: Supplementary Figure 2 — The BCVA examined at baseline, 12 and 24 months using T&E and PRN regimen for SRF permissive patients (left) and PCV patients (right). [file Figure_2.jpg]

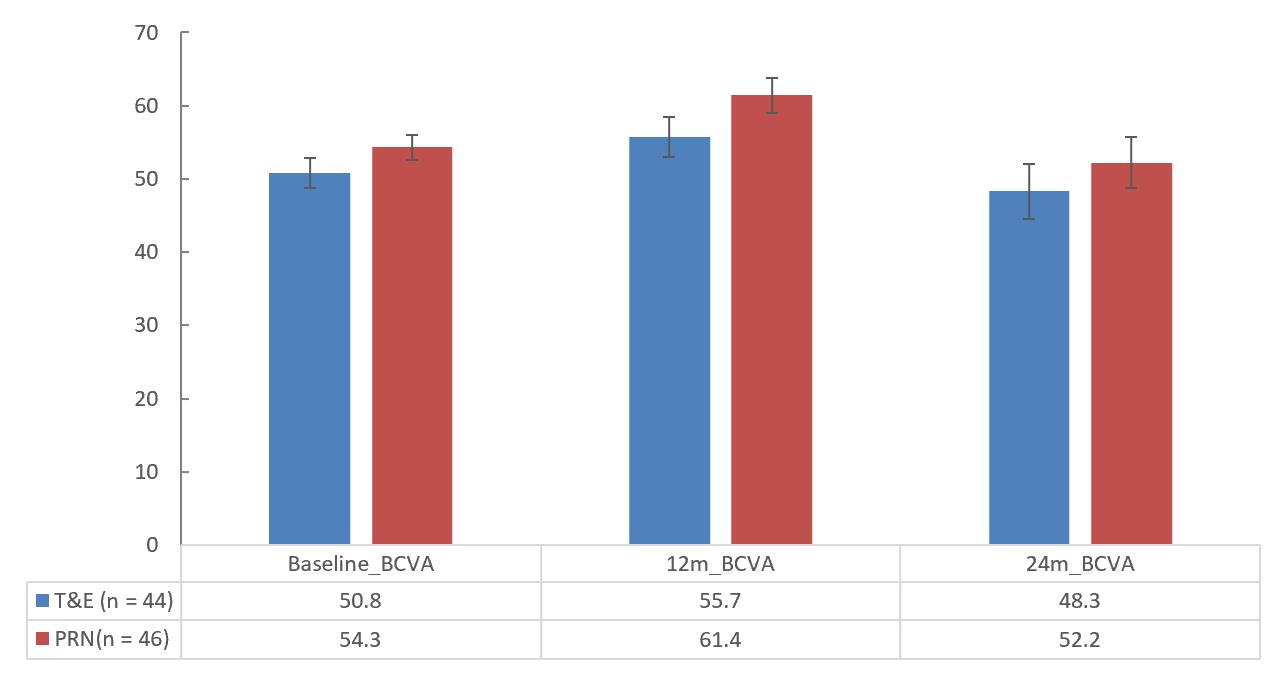

Supplement: Supplementary Figure 3 — Comparisons of BCVA observed from patients treated with T&E and PRN regimen (only patients who completed the 24-months follow-up were included, n = 90). [file Figure_3.jpg]
