# Supplementary material for: A Randomized, Controlled Trial of Treat-and-Extend vs. Pro Re Nata Regimen for Neovascular Age-Related Macular Degeneration
Source: Front Med (Lausanne). 2022 Jun 20;9:852519. doi: 10.3389/fmed.2022.852519 (PMC9251380; doi:10.3389/fmed.2022.852519)
Supplement: Supplementary file 4 [file Data_Sheet_1.docx]

**eTable 1. Proportion of Patients With Gain or Loss of 15 or More ETDRS Letters at 24 Months**

| **Outcomes** | **T&E** | **PRN** | ***P* Value** |
| --- | --- | --- | --- |
| Letters gained ≥ 15, % of patients | 20.3 | 27.8 | 0.299 |
| Letters lost ≥ 15, % of patients | 10.1 | 12.5 | 0.659 |

Abbreviations: ETDRS, Early Treatment of Diabetic Retinopathy Study; T&E, treat-and-extend; PRN, pro re nata.

**eTable 2. The BCVA outcomes in Subgroup analysis**

| **Subgroup** | **BCVA (ETDRS letters)** | **T&E** (Mean± SD) | **PRN** (Mean± SD**)** | **Difference (95% CI)** |
| --- | --- | --- | --- | --- |
| PCV |  | *n* = 18 | *n* = 28 |  |
|  | Baseline BCVA | 54.6 ± 11.5 | 52.5 ± 14.5 | 2.075 (-6.084 ~ 10.234) |
|  | Mean change in BCVA at 12m | 2.2 ± 23.2 | 9.4 ± 12.8 | -7.135 (-17.820 ~ 3.550) |
|  | Mean change in BCVA at 24m | -1.2 ± 23.6 | 1.6 ± 18.5 | -2.865 (-15.391 ~ 9.661) |
| SRF permissive |  | *n* = 22 | *n* = 27 |  |
|  | Baseline BCVA | 58.7± 10.5 | 55.5 ± 12.3 | 3.245 (-3.430 ~ 9.922) |
|  | Mean change in BCVA at 12m | 7.9 ± 12.7 | 8.3± 9.4 | -1.168(-7.536 ~ 5.200) |
|  | Mean change in BCVA at 24m | 0.7 ± 13.1 | 3.5 ± 10.0 | -2.800(-9.447 ~ 3.848) |
| Naive |  | *n* = 49 | *n* = 56 |  |
|  | Baseline BCVA | 52.8 ± 13.0 | 51.8 ± 14.3 | 1.028 (-4.295 ~ 6.352) |
|  | Mean change in BCVA at 12m | 6.2 ± 18.4 | 11.2 ± 13.0 | -4.954 (-11.051 ~ 1.143) |
|  | Mean change in BCVA at 24m | 4.6 ± 18.6 | 6.2 ± 16.4 | -1.625 (-8.383 ~ 5.133) |
| Nonnaive |  | *n* = 19 | *n* = 16 |  |
|  | Baseline BCVA | 53.5 ± 16.6 | 52.4 ± 11.8 | 1.036 (-9.039 ~ 11.111) |
|  | Mean change in BCVA at 12m | 5.7 ± 16.8 | 7.8 ± 10.3 | -2.066 (-11.881 ~ 7.749) |
|  | Mean change in BCVA at 24m | 2.4 ± 15.8 | 1.4 ± 16.2 | 0.993 (-10.034 ~ 12.021) |

Abbreviations: ETDRS, Early Treatment of Diabetic Retinopathy Study; BCVA, Best-Corrected Visual Acuity; T&E, treat-and-extend; PRN, pro re nata.

**eTable 3. The 12 months and 24 months BCVA change from baseline in on-treatment-analyses**

| BCVA Mean change | **T&E** | **PRN** | **Differrence (95% CI)** |
| --- | --- | --- | --- |
|  | *n* = 44 | *n* = 46 |  |
| By the end of 24 months | 3.0± 18.3 | 1.2 ± 17.2 | 1.783(-5.676~9.243) |
|  | *n* = 52 | *n* = 59 |  |
| By the end of 12 months | 4.8±17.3 | 8.8±12.3 | -4.047(-9.653~1.559) |

Abbreviations: BCVA, Best-Corrected Visual Acuity; T&E, treat-and-extend; PRN, pro re nata.

**eTable 4. Centre Subfield Thickness Change From Baseline to 24 Months**

|  | **T&E (*n* = 69)** | **PRN (*n* = 72)** | ***P* value** |
| --- | --- | --- | --- |
| Mean change in CST at 12m (μm) | -168 ± 229 | -257 ± 211 | 0.049 |
| Mean change in CST at 24m (μm) | -180 ± 165 | -247 ± 230 | 0.135 |

Abbreviations: CST, Centre subfield thickness; T&E, treat-and-extend; PRN, pro re nata.
